# Blood Eosinophils and Pulmonary Rehabilitation in COPD

**DOI:** 10.1155/2021/7449527

**Published:** 2021-11-05

**Authors:** Jafar Aljazeeri, Abdullah Sakkat, Nima Makhdami, Rayyan Almusally, Frederick Morfaw, Andrew McIvor

**Affiliations:** ^1^University of Pittsburgh Medical Center, Pennsylvania, USA; ^2^Drexel University College of Medicine, Pennsylvania, USA; ^3^McMaster University, Department of Medicine, Division of Respirology, Hamilton, Canada; ^4^Firestone Institute for Respiratory Health, St Joseph Healthcare, Hamilton, Canada; ^5^Imam Abdulrahman Bin Faisal University, Dammam, Saudi Arabia; ^6^McMaster Univerisity, Department of Health Research Methods, Evidence and Impact, Hamilton, Canada

## Abstract

**Background:**

Blood eosinophils predict the response to therapy, risk of exacerbation, and readmission in COPD. This study investigates whether blood eosinophils predict pulmonary rehabilitation (PR) outcomes in COPD.

**Methods:**

We categorized patients into eosinophilic (blood eosinophils ≥300 cells/ml) or noneosinophilic (<300 cells/ml). In a retrospective design, we compared changes within and between the two groups on BODE index, 6-minute walk test (6MWT), FEV1, and mMRC dyspnea scale.

**Results:**

Of 206 patients enrolled, 176 were included for analysis; 90 were eosinophilic. BODE index improved in both groups: (MD −1.25; 95% CI (−0.45, −4.25), *P* ≤ 0.001) in the eosinophilic and (MD −1.33; 95% CI (−1.72, −0.94), *P* ≤ 0.001) in the noneosinophilic, but a higher BODE index remained in the eosinophilic (4.98); adjusted mean change (*β*): 0.7 (95% CI (0.15, 1.26), *P*=0.01). 6MWT improved by 29.3 m in the eosinophilic (95% CI (14.2, 44.4), *P* ≤ 0.001) vs. 115.1 m in the noneosinophilic (95% CI (−30.4, 260.6), *P*=0.12). FEV1 did not change in the eosinophilic (MD −0.6; 95% CI (−2.64, 1.48), *P*=0.58), but improved by 2.5% in the noneosinophilic (MD 2.5; 95% CI (0.77, 4.17), *P*=0.005). There were no significant between-group differences in 6MWT and FEV1; adjusted mean changes (*β*) were −9.69 m (95% CI (−39.51, 20.14), *P*=0.52) and −2.31% (95% CI (−5.69, 1.08), *P*=0.18), respectively. There were no significant within- or between-group changes in the mMRC scale.

**Conclusion:**

Although PR improves the BODE index in both eosinophilic and noneosinophilic COPD, a higher eosinophil count (≥300 cells/ml) is associated with a higher (worse) BODE index. Blood eosinophils may predict PR outcomes.

## 1. Introduction

Chronic obstructive pulmonary disease (COPD) is a progressive disease characterized by significant airflow limitation, lung destruction, and associated with chronic airway inflammation [1]. The pattern of inflammation in COPD is typically neutrophilic [2]; however, a subgroup of patients demonstrates high eosinophil levels, either in sputum or blood [3]. Although patients with eosinophilic phenotype exhibit the greatest response to corticosteroid therapy [4], several prospective studies suggest that eosinophilic inflammation is associated with an increased risk of exacerbation, readmission, and poor clinical outcomes [5–8].

The standard management of COPD consists of pharmacological and nonpharmacological treatments. Several recent studies have looked at the role of blood eosinophils in pharmacological treatment. For example, a post hoc analysis of 3 randomized trials showed that blood eosinophils can predict the effect of inhaled corticosteroids (ICS) in preventing future exacerbations when added to maintenance bronchodilator therapy [9]. Another pooled analysis of 2 trials found that adding roflumilast to combined bronchodilator therapy may reduce the risk of exacerbation in severe COPD with a greater benefit in patients with higher baseline eosinophil count [10]. Besides predicting the treatment effect, previous cohort studies have demonstrated that blood eosinophils can also predict future exacerbations [8, 11]. On the other hand, scarce data, if any, exist as to whether blood eosinophils predict nonpharmacological treatment.

Pulmonary rehabilitation (PR) is a comprehensive integrated nonpharmacological intervention, a central component of the management of COPD, and recommended by most professional organizations and guidelines [1, 12]. PR improves health status, dyspnea, and exercise tolerance and reduces hospitalization [13]. We also learned from prior studies that objective measurements, such as a 6-minute walk test (6MWT) and forced expiratory volume in 1 second (FEV1), improve after PR [14, 15]. Beyond that, the ultrasound measurements of diaphragmatic mobility have shown a promising role of this noninvasive modality and may predict outcomes pre and post-PR in COPD patients [16]. However, it remains unknown whether a specific COPD phenotype (eosinophilic or noneosinophilic) benefits better than the other. Thus, we aimed to study the relationship between blood eosinophils and different measurable PR outcomes in COPD.

## 2. Methods

### 2.1. Study Design and Population

This is a retrospective observational cohort study. We extracted the study data from the electronic records database located in a university-affiliated tertiary center, the Firestone Institute for Respiratory Health, McMaster University, Hamilton, Canada. We reviewed all patients admitted into the 6-week inpatient PR program between January 2010 and May 2016. This project was approved by the institutional ethics committee (Hamilton Integrated Research Ethics Board) and conducted according to the Declaration of Helsinki.

We included participants who were ≥ 40 years of age, enrolled in the inpatient PR program with a primary documented diagnosis of COPD, based on symptoms, spirometry results, and the standard definition according to the Global Initiative for Chronic Obstructive Lung Disease and discharged alive from the program.

Exclusion criteria upon candidacy assessment were age <40 years, absence of fixed airflow limitation on spirometry results, never smokers, and enrollment for other obstructive pulmonary diseases (e.g. asthma and bronchiectasis), interstitial lung disease, pulmonary vascular disease, or prelung transplantation.

### 2.2. Data Collection

We collected baseline characteristic data: age, sex, smoking status, vaccination history, COPD therapy, home oxygen use, partial pressure of carbon dioxide (pCO2) level, use of walking aids, and blood eosinophil count level. Data for outcomes were collected at baseline and postrehabilitation and included: the body mass index (BMI)/airflow obstruction/dyspnea/exercise capacity (BODE) index, distance walked in the 6-minute test (6MWT), the spirometry results of the force expiratory volume in 1 second (FEV1), and modified Medical Research Council (mMRC) dyspnea scale.

### 2.3. Study Variables and Outcomes

The principal independent variable was blood eosinophil cell count available at the time of admission or within the past 6 months of enrollment into the inpatient PR program. The cutoff (≥300 cells/ml) has previously shown a strong association with the risk of exacerbation and all-cause mortality in large epidemiological studies and post hoc analyses of clinical trials [9]. Thus, we considered this level to indicate the eosinophilic group. The outcomes of interest were the changes in the BODE index, 6MWT, FEV1, and mMRC dyspnea scale, within and between groups.

### 2.4. Statistical Analyses

We categorized patients into eosinophilic (≥300 cells/ml) and noneosinophilic groups (<300 cells/ml). We compared changes in study variables between the two groups. We used the paired sample *t*-test to estimate the within-group changes (mean postrehabilitation minus mean at baseline) on the BODE index, 6MWT, and FEV1. The comparison of between-group differences in these outcomes was done by using multivariable linear regression. The independent variable was the eosinophil category, while the different outcomes (BODE index, 6MWT, and FEV1) served as the dependent variables. For the mMRC dyspnea scale, we categorized this outcome into a binary outcome (improved or not improved). We used logistic regression following PR between the two groups. All variables were adjusted for age, gender, current smoking status, history of smoking, the interaction between current smoking status and history of smoking, vaccination history, COPD treatment regimen, BMI, pCO_2_ retainer status, home O_2_ therapy status, use of walking aids, and the respective baseline value for each outcome. All analyses were performed using SPSS version 25.0 [17].

## 3. Results

**Figure 1:**
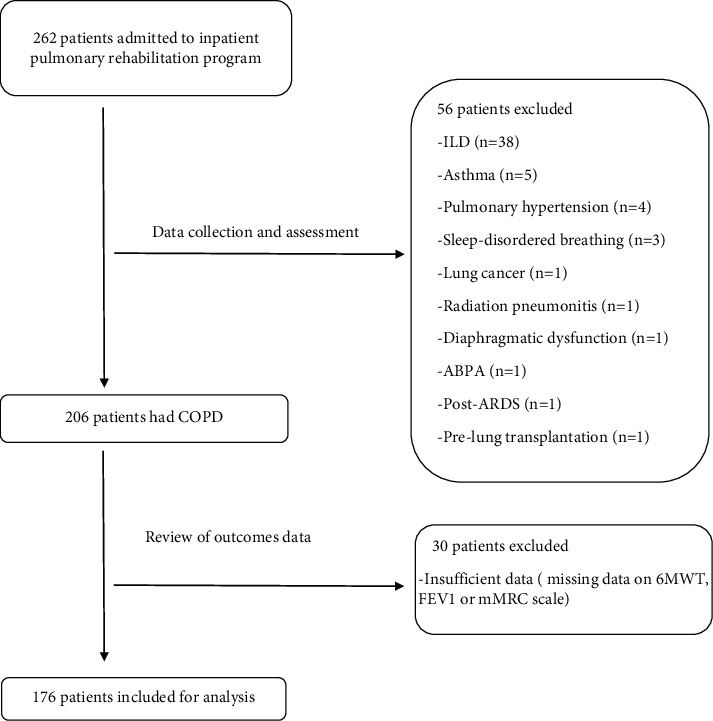
Flowchart of patients' enrollment process.

A total of 262 patients were admitted to the inpatient PR program during the data collection period between January 2010 and May 2016. Of them, 206 candidates met the inclusion criteria. Reasons for exclusion are shown in Figure 1. 176 patients had complete data, thus included for analysis. 90 patients (51.1%) were eosinophilic (blood eosinophils ≥300 cells/ml). Patients' baseline characteristics are given in Table 1.

### 3.1. BODE Index

The average baseline BODE index was 5.83 in the eosinophilic group, whereas 5.50 in the noneosinophilic group. In both groups, BODE index improved significantly post-PR (mean difference (MD) −1.25; 95% CI (−0.45, −4.25), *P* <0.001) in the eosinophilic group versus (MD −1.33; 95% CI (−1.72, −0.94), *P* <0.001) in the noneosinophilic group (Table 2). Post-PR analysis showed a higher mean BODE index in the eosinophilic group (4.98) when compared with noneosinophilic (4.17). After a fully adjusted analysis, the between-group comparison (eosinophilic vs. noneosinophilic) in the BODE index differed significantly post-PR (adjusted mean change (*β*), 0.7 (95% CI (0.15–1.26)), *P*=0.01) (Table 3).

### 3.2. 6-Minute Walk Test

The average baseline total walked distanced in the eosinophilic group was 240 meters (*m*) compared with 250 m in the noneosinophilic group. Post-PR, 6MWT improved by 29.2 m (CI (14.2, 44.4), *P* ≤ 0.001) in the eosinophilic group and by 115.1 m (CI (−30.4, 260.6), *P*=0.12) in the noneosinophilic group (Table 2). No significant between-group changes were in 6MWT: −9.69 m (95% CI (−39.51, 20.14), *P*=0.52) (Table 3).

### 3.3. FEV1

The mean baseline FEV1 was 35.6% in the eosinophilic group versus 36.8% in the noneosinophilic group. No significant change in the mean FEV1 post-PR among the eosinophilic group (MD −0.6; 95% CI (−2.64, 1.48), *P*=0.58). However, we observed a mild increase of the mean FEV1 by 2.5% in the noneosinophilic group (MD 2.5; 95% CI (0.77, 4.17), *P*=0.005) (Table 2). FEV1 did not differ in between-group comparison; adjusted mean change (*β*) is −2.31 (95% CI (−5.69, 1.08), *P*=0.18) (Table 3).

### 3.4. mMRC

The mean mMRC dyspnea scale was nearly similar between both groups at baseline. Neither group showed improvement post-PR; 86.6% of the eosinophilic and 86.0% of the noneosinophilic had no improvement of the mMRC scale (Table 2). The odds of improvement in the mMRC scale between both groups were not significant; adjusted OR was 1.21 (95% CI (0.17, 8.13), *P*=0.84) (Table 3).

## 4. Discussion

The main finding of this study is that COPD with eosinophilic inflammation (defined as blood eosinophil count ≥300 cells/ml) is associated with less favorable pulmonary rehabilitation (PR) outcomes when compared with their counterpart noneosinophilic (<300 cells/ml). Although improved in both groups post-PR, we found a higher mean BODE index in the eosinophilic group. This impact of eosinophil counts on the BODE index is consistent after fully adjusted analyses on multiple variables. Other outcomes (6MWT, FEV1, and mMRC) did not differ between both groups, but we observed a trend of better performance in the noneosinophilic group.

The BODE index is a validated assessment tool that has a prognostic value for the risk of exacerbations and mortality in COPD [18, 19]. The change in the BODE index predicts survival better than any changes of its individual components (mMRC, FEV1, 6MWT, and BMI)—an increase of BODE index ≥1 is associated with increased mortality [20]. In our study, the BODE index improved in both groups by more than 1, and likely, both groups have benefited from the PR program. However, the degree of improvement was higher in the noneosinophilic patients, leaving questions on why such improvement is not seen in the eosinophilic patients and how to improve their PR outcomes? Regardless, whether blood eosinophils are independent factors per se or whether eosinophilic inflammation is controlled or not, more attention is needed for this subgroup of COPD. Recently, many studies focused on targeted management of eosinophilic inflammation. For example, data from randomized controlled trials (RCTs) concluded that adding ICS is beneficial in controlling COPD with blood eosinophil count (≥300 cells/ml) and reducing future exacerbations [9, 21]. Another data from 2 RCTs found that antiinterleukin 5 therapy (mepolizumab) might reduce the annual rate of moderate or severe exacerbations in patients with COPD and eosinophilic phenotype [22]. Following the results of our study, we might be able to improve PR outcomes if we control the eosinophilic inflammation. Indeed, we need further studies to understand if adding therapies to control eosinophilic inflammation improves PR outcomes in COPD with higher blood eosinophils.

While there are no significant between-group differences in other outcomes (FEV1, 6MWT, and mMRC), a greater within-group performance is seen in the noneosinophilic group. For instance, the mean FEV1 post-PR increased by 2.5%, which mirrors the expected change in FEV1 from previous data [14]. The 6MWT improved by 115 m in the noneosinophilic group but was not statistically significant. This magnitude (115 m) is probably a skewed data distribution and is likely driven by the heterogeneity/wide confidence interval in this group. In contrast, the 6MWT in the eosinophilic group improved significantly post-PR but only by 29.2 m. This resembles the minimal clinically important difference for the change in 6MWT based on previous consensus [23, 24], yet less than the average of improved distance (62 m) after COPD exacerbation estimated in a meta-analysis of 13 studies [15].

In this study, most patients in both groups experienced no improvement in their mMRC dyspnea scale. We attribute this finding to the insensitivity of the mMRC scale to change in response to therapeutic intervention, a common limitation of the mMRC scale, and particularly seen in moderate to severe COPD [25, 26]. Our cohort represents mostly severe COPD, with an average baseline FEV1 of 36%, and more than half of them were on home O_2_ therapy; thus, the mMRC scale expectedly tends to congregate between 3 and 4. We know from previous studies that perceived dyspnea usually improves following the PR program irrespective of the measurement tool used or the baseline mMRC scale [27, 28]. Eventhough the mMRC scale is a simple tool and widely used for referral to PR, it is only appropriate for discriminative properties and less useful for evaluative properties when compared with multidimensional tools such as the Chronic Respiratory Disease Questionnaire (CRQ) and the St. George's Respiratory Questionnaire (SGRQ) [29]. Hence, practice guidelines recommend rather using a multidimensional tool for evaluating dyspnea outcomes after PR instead of using the mMRC scale [12].

Our study has several merits. It is the first, to our knowledge, that studied the association of a biomarker (blood eosinophils) with pulmonary rehabilitation. This permits a wider vision of care when providing nonpharmacological interventions to COPD patients. Besides, we fully adjusted important variables that might have influenced the targeted outcomes to minimize potential confounders. Nonetheless, the study is retrospective in nature, and the results should be interpreted with caution. It was conducted in a single center, which may restrict generalizability. Moreover, additional data on clinical follow-ups and long-term assessments were missing. Last, the exact doses of ICS therapy were not available, but we know that a large proportion of patients were using ICS, and they were nearly similar (80–90%) in both groups. We acknowledge that this might indicate an unclear status on whether the eosinophilic inflammation is adequately optimized. In such cases, where eosinophilic status is readily needed, we recommend that respiratory physicians consider using a point-of-care device to assess the blood eosinophil count during the initial PR visit, as this quick tool has shown reliable measurements [30]. Our study emphasizes that more attention to this subgroup of COPD is warranted. Future studies with larger samples are needed to clarify if blood eosinophils independently predict PR outcomes or if the results of this study merely represent under management of this subgroup. In the end, the results represent a motive for a better understanding of the role of blood eosinophils beyond the response to pharmacological interventions and promote future initiatives to enhance the optimal management of this progressively disabling disease.

## 5. Conclusion

In COPD, a blood eosinophil count ≥300 cells/ml is associated with less favorable pulmonary rehabilitation outcomes—substantially, this level is associated with a higher BODE index. The findings reaffirm the importance of appropriately phenotyping and managing COPD. Blood eosinophils may predict PR outcomes in COPD.

## Figures and Tables

**Table 1:** Baseline characteristics of the study participants.

Variables	Eosinophil count ≥300 (*n* = 90)	Eosinophil count <300 (*n* = 86)
Age (year), mean ± SD	67.4 ± 8.9	67.7 ± 12.0
BMI (kg/m^2^), mean ± SD	24.4 ± 7.3	26.6 ± 7.5
Gender, *n* (%)		
Female	42 (46.7)	37 (43.0)
Male	48 (53.3)	49 (57.0)
Current smoking status, *n* (%)		
Smoker	13 (14.4)	10 (11.6)
Pack-year	50.1 (25.1)	58.7 (23.0)
Vaccination history, *n* (%)		
Influenza vaccine	79 (87.8)	80 (93.0)
Pneumococcal vaccine	66 (73.3)	54 (62.8)
COPD treatment, *n* (%)		
SABA	87 (96.7)	79 (91.9)
SAMA	6 (6.7)	5 (5.8)
ICS/LABA	80 (88.9)	70 (81.4)
LAMA	81 (90.0)	72 (83.7)
PDE4 inhibitor	3 (3.3)	2 (2.3)
Systemic corticosteroids	19 (21.1)	22 (25.6)
Macrolides	4 (4.4)	5 (5.8)
Home O_2_ therapy		
Yes	58 (64.4)	45 (52.4)
Walking aids, *n* (%)		
Yes	54 (60.0)	39 (45.3)
pCO_2_ retainer, *n* (%)		
Yes	39 (43.3)	34 (39.6)
CO_2_ level (mmHg), mean ± SD	45.2 ± 8.1	45.9 ± 10.1
BODE index, mean ± SD	5.8 ± 2.1	5.5 ± 2.1
6MWT (m), mean ± SD	242.9 ± 105.9	247.3 ± 110.4
FEV1 (%), mean ± SD	35.5 ± 30.1	36.9 ± 16.7
Dyspnea mMRC scale, *n* (%)		
0	3 (3.5)	2 (2.5)
1	6 (7.1)	11 (13.6)
2	33 (38.8)	28 (32.6)
3	18 (21.2)	22 (25.6)
4	24 (28.2)	18 (20.9)

Data are presented as mean ± SD (standard deviation), *n* (number), or % (percentage). Pack-year is calculated by multiplying the number of packs of cigarettes smoked per day by the number of years the person has smoked. SABA, short-acting *β*-agonist; SAMA, short-acting muscarinic antagonist; ICS/LABA, inhaled corticosteroid/long-acting *β*-agonist; LAMA, long-acting muscarinic antagonist; PDE4, phosphodiesterase type 4; O_2_, oxygen; pCO_2_, partial pressure of carbon dioxide; mmHg, millimeter mercury; BODE, body mass index, airflow obstruction, dyspnea, and exercise; 6MWT, 6-minute walk test; m, meter; mMRC, modified Medical Research Council.

**Table 2:** Within-group change on outcomes postpulmonary rehabilitation.

Outcome	Eosinophils	Baseline, mean	Post-PR, mean	Mean difference (95% CI)	*P* value
BODE index	≥300	5.83	4.58	−1.25 (−0.45, −4.25)	<0.001
	<300	5.50	4.17	−1.33 (−1.72, −0.94)	<0.001
6MWT (m)	≥300	240.3	269.5	29.2 (14.2, 44.4)	<0.001
	<300	250.4	365.5	115.1 (−30.4, 260.6)	0.12
FEV1 (%)	≥300	35.6	35.0	−0.6 (−2.64, 1.48)	0.58
	<300	36.8	39.3	2.5 (0.77, 4.17)	0.005
			No improvement (%)	Improved (%)	
mMRC scale^*∗*^	≥300		86.6	13.3	n/a
	<300		86.0	14.0	n/a

PR, pulmonary rehabilitation; CI, confidence interval; BODE, body mass index, airflow obstruction, dyspnea, and exercise capacity; 6MWT, 6-minute walk test; m, meters; FEV1, forced expiratory volume in 1 second; mMRC, modified Medical Research Council; n/a, not applicable. ^*∗*^mMRC change measured as a proportion (%).

**Table 3:** Between-group change on outcomes postpulmonary rehabilitation.

Outcome	Unadjusted *β* (95% CI)	*P* value	Adjusted *β* (95% CI)	*P* value
Change in BODE index.	0.68 (0.04, 1.30)	0.04	0.70 (0.15, 1.26)	0.01
Change in 6MWT (m)	−15.02 (−44.21, 14.16)	0.31	−9.69 (−39.51, 20.14)	0.52
Change in FEV1 (%)	−2.31 (−6.03, 1.41)	0.22	−2.31 (−5.69, 1.08)	0.18

	Unadjusted OR (95% CI)		Adjusted OR (95% CI)	
Change in mMRC scale	0.95 (0.40, 2.24)	0.91	1.21 (0.17, 8.13)	0.84

CI, confidence interval; BODE, body mass index, airflow obstruction, dyspnea, and exercise capacity; 6MWT, 6-minute walk test; FEV1, forced expiratory volume in 1 second; OR, odds ratio. Variables adjusted for include age, gender, smoking status, history of smoking, the interaction between current smoking status and history of smoking, vaccination history, COPD treatment regimen, BMI, pCO_2_ retainer status, home o_2_ therapy status, use of walking aids, and the respective baseline values for each outcome.

## Data Availability

The data used to support the findings of this study are available from the corresponding author upon request.
