# Correction to “Blood Eosinophils and Pulmonary Rehabilitation in COPD”

**DOI:** 10.1155/carj/9824978

**Published:** 2026-03-04

J. Aljazeeri, A. Sakkat, N. Makhdami, R. Almusally, F. Morfaw, and A. McIvor, “Blood Eosinophils and Pulmonary Rehabilitation in COPD,” *Canadian Respiratory Journal*, 2021, https://doi.org/10.1155/2021/7449527.

In the article, incorrect units were used when describing the values of the blood eosinophil count. The correct units should be cells/µL throughout the article.

We apologize for this error.

